# 

**Published:** 1884-07

**Authors:** 


					﻿BOOKS AND PAMPHLETS RECEIVED.
Pathology and Treatment of Gonorrhoea, by J. L. Milton, Senior Sur-
geon to St. John’s Hospital for diseases of the skin. London : fifth
edition. William Wood& Co., New York, 1884.
Legal Medicine, by Charles Meymott Tidy, M. B., F. C. S. London.
Vol. 3. William Wood & Co., New York, 1884.
Diseases of the Heart, by Constantine Paul. Translated from the
French. William Wood & Co., New York, 1884.
Sexual Neurasthenia, its hygiene, causes, symptomsand treatment,
by George M. Beard, A. M., M. D. Edited by A. D. Rockwell, 757
Broadway. E. B. Treat, New York, 1884.
Elementary Principles of Electro-Therapeutics, for the use of physi-
cians and students, byC. M. Haynes, M. D. Published by the Mc-
Intosh Galvanic and Faradic Battery Company, Chicago, Ill., 1884.
Iodoform in Dental Surgery, by C. F. W. Bodecker, D. D. S., M. D. S.,
New York. Reprint from the Independent Practitioner of March,
1884.
Drugs and Medicines of North America, a quarterly, by J. U. A G. C.
Lloyd, Cincinnati.
Hooper's Physician's' Vade Mecum. A manual of the principles and
practice of physic, with an outline of general Pathology, Thera-
peutics and Hygiene. Tenth Edition—Revised by William Augus-
tus Guy, M. B Cantab—F. R. S. and .John Harley, M. D. Bond—
F. L. S. Vol. 1, William Wood & Co., New York, 1884.
Practical Mutual of Obstetrics, Dr. E. Verrier, first American edi-
tion, with revisions and ann Nations, by Edward L. Partridge, M.
D. William Wood & Co., New York, 1884.
Health Hints for Travelers,-by John C. Sunberg, M. D. D. G.
Brinton, Philadelphia, 1884.
Address on Practical Medicine, by John V. Shoemaker, A. M., M. D.
chairman of Section of Practice, etc. American Medical Associa-
tion, 1884.
Annual Announcement Medical College of Georgia. Augusta,
1884-85.
Annual Announcement of the Medical Department of the Willam-
ette University, Portland, Oregon, 1884-85.
Abscess of Maxillary Sinus, caused by Syphilis. By Jno. D. S. Davis,
M. D., Birmingham, Ala.
Malarial Amblyopia, by John D. S. Davis, M. D., Birmingham,
Ala.
Annual Announcement of Medical Department of Dartmouth Col-
lege, Hanover, N. H., 1884.
Annual Announcement Woman’s Medical College of Pennsylvania,
Philadelphia, 1884-85.
PUBLISHERS’ ANNOUNCEMENT.
We send a number of this issue of The Journal to physicians
who are not subscribers. As as inducement to get them to sub-
scribe we will, for the next thirty days, enter all subscriptions at
$1.50 per annum. This offer is open to all subscribers whose time
has or is about to expire—provided the money is sent by August 1st.
This is a special offer, to last only for the time specified, and is made
to induce those to whom sample copies are sent to become subscribers.
Jas. P. Harrison & Co., Publishers.
OUR ADVERTISERS.
Doliber, Goodale & Co., Boston.—These gentlemen are the proprie-
tors of the justly celebrated Mellin’s Food for infants and invalids.
We consider this food superior to any artificial food manufactured.
See their advertisement on fourth page of cover and write them for
sample bottle, mentioning the Journal, and it will be sent you free.
Cincinnati Sanitarium.—This is a private hospital for the insane,
situated at College Hill, Ohio. It has been in successful operation
for a number of years, and has had patients from all parts of the
country.
Our physicians having patients that need the attention given at
such institutions would do well to correspond with the superintend-
ent before sending them elsewhere. See the advertisement.
Medical Department University of Louisiana.-—This old and popular
institution of learning has an advertisement in this issue of the
Journal There is no institution of learning in the United States
that stands higher than does this one. We predict for it the com-
ing session a large class. See the advertisement, to be found else-
where, and write to the Dean for the annual announcement.
H. H. Burrington, M. D., Providence.—This gentleman has made a
specialty for a long time of the manufacture of gynecological appli-
ances. In another part of the .Journal will be found his adver-
tisement, with a cut of his pessary, for which he has a large sale.
See his advertisement and write him for any information you may
desire, he will cheerfully give it.
East Tennessee, Virginia & Georgia Railroad.—This popular road
has an advertisement elsewhere in the Journal It is the direct
short line to all Southern points. They are now selling excursion
tickets from Atlanta to the beautiful Cumberland Island for S10.00
round trip.
Does Advertising Pay.—Messrs. Magnus & Hightower, of Atlanta,
recognizing the value of judicious advertising, gave us a page ad-
vertisement for our last Journal, making a contract for the year.
They inform us now that the profits on their sales from orders re-
ceived from the one issue of their advertisement will pay their con-
tract for the year. This is very gratifying to us, for we desire to
see these young gentlemen succeed, and we know that their success
is assured when they have, in addition to their large city trade, an
outside trade that older houses might be proud of. We repeat what
we have had occasion to say before, that physicians cannot buy
drugs, chemicals, surgical instruments, etc., cheaper anywhere than
from this house.
NOTICE.
Original communications solicited. When articles require illustration the
engravings will be furnished by the publishers free of charge.
Subscription : $2.50 a year.
Subscribers will please not send us money by Postal Notes. Remit by
Express, Draft, Post-office Order, or Registered Letter.
Address all letters and books, and make all drafts payable to
The Atlanta Medical and Surgical Journal,
Care Jas. P. Harrison & Co., Atlanta, Ga.
				

